# Supplementary material for: Adherence to practice parameters in Medicare beneficiaries with amyotrophic lateral sclerosis
Source: PLoS One. 2024 Jun 3;19(6):e0304083. doi: 10.1371/journal.pone.0304083 (PMC11146737; doi:10.1371/journal.pone.0304083)
Supplement: Attachment — Submitted filename: ALS Utilization - Response to Reviewers.docx [file pone.0304083.s002.docx]

**PONE-D-23-13322 - Adherence to practice parameters in medical beneficiaries with amyotrophic lateral sclerosis.**

Dear Dr. Chidi Chima,

We appreciate the opportunity to revise our manuscript (PONE-D-23-13322) in response to reviewers’ comments on our manuscript entitled “Adherence to practice parameters in medical beneficiaries with amyotrophic lateral sclerosis.” We have addressed all reviewer comments. The major change to the manuscript is the inclusion of a sensitivity analysis that excludes potential amyotrophic lateral sclerosis (ALS) mimics. This sensitivity analysis did not reveal a major difference in our findings, further strengthening our paper. We have supplied these results as Supplemental tables 1-2. We respond to your and the reviewers’ comments in point form below:

**Reviewer 1**

1. *Kindly elaborate a little bit on the "right to access to neurological care and treatment to larger groups of US residents", especially minority population groups, the elderly, and other disadvantaged population groups.*

**Response:** We appreciate the opportunity to be able to further elaborate on this important topic.

Pg #20-21, Line 329-338. “Our study highlights the importance of receiving specialist care for ALS patients and provides evidence that may guide health policies and education efforts in the U.S. Efforts such as ensuring early referral and access to neurologist care could emerge as significant interventions. The focused training on neurologic disease during residency and accumulated clinical experience with ALS likely improved adherence to the practice parameters. Neurologists may also be more successful in the early recognition and management of common ALS-associated comorbidities such as need for feeding tube or NIV. Unfortunately, due to a shortage of neurologists this may not always be possible, thus a more systematic approach to education of non-neurologic providers on basic management and focused on adherence to the practice parameters may improve ALS care. This is of critical importance as the aged population continues to increase, the treatment disparities presented here may continue to worsen without efforts to mitigate them, in particular for black and disadvantaged beneficiaries.”

**Reviewer 2**

1.  *Kindly re-evaluate your data to see " if there is a possibility that there are some patients who have ALS diagnosis at first but have other subsequent diagnoses, especially for the patients who didn’t receive the diagnosis from a neurologist." If this is so, consider excluding this category of patients from the analysis, or provide justification and potential impact of their inclusion in the study results.*

**Response:** We performed sensitivity analyses that exclude potential ALS mimic-codes based on the literature. We chose to use the most inclusive definition of a mimic to perform a sensitivity analysis, with an alternative case definition that contained 6,888 beneficiaries (or a 19.7% sample reduction). Despite this 19.7% reduction, there were no significant changes in our findings. Supplemental tables 1-2 highlight the robustness of our cohort and study findings.

Pg #5, Line 92-101. “As an alternative case definition for sensitivity analyses, we excluded those patients that had an ICD-9 code consistent with a potential ALS mimic [17] after they had received a 335.2x code to minimize the possibility of inappropriately miss-accounting differences in practice parameter utilization in those patients whose diagnosis changed after the initial ALS diagnosis (Supplemental Tables 1-2). These potential mimics included structural spinal pathologies, myasthenia gravis and subtypes, polyneuropathies, and spinal muscular atrophies. We did not exclude patients with a code for another MND as these diseases have a common phenotype and may be difficult to fully differentiate [18]. In addition, based on prior administrative data algorithms [13], and the reported low misclassification by neurologist [17, 19, 20], we retained as true ALS cases those patients who received an ALS code from a neurologist. Utilizing this alternative case definition, we identified 6,888 incident ALS cases for sensitivity analysis.”

Pg #6, Line 138-140. “We performed a sensitivity analysis with our alternative case definition to assess the effects of excluding those beneficiaries with a potential ALS mimic diagnosis.”

Pg #11, Line 208-210. “Additionally, in sensitivity analysis our findings were overall unchanged when beneficiaries with potential ALS mimics were excluded from our cohort. (Supplemental Tables 1-2)”

Pg #21, Line 356-358. “Furthermore, in sensitivity analysis excluding potential ALS mimic, despite a reduction of up to 20% of our sample, our key findings were unchanged, highlighting the robustness of our findings and cohort.”

Supplemental Tables 1-2, footnotes: Sensitivity analysis limited to beneficiaries with an ALS code from a neurologist and those with an ALS code but without a potential ICD-9-CM ALS mimic code at any time after their initial ALS diagnosis. Potential diagnosis mimic codes include: Structural spinal pathology (spondylosis and allied disorder – 721.0-4x, Spinal stenosis in cervical region – 723.0, and spinal stenosis of other regions – 724.0x), Myasthenia gravis and subtypes (358.0x, 358.1, 358.3x, 358.8, 358.9), Polyneuropathy (Polyneuropathy in other diseases classified elsewhere, including Multifocal motor neuropathy – 357.4, Diabetic Polyneuropathy – 357.2, Hereditary spastic neuropathy – 356.x, Chronic Inflammatory Demyelinating Polyneuropathy – 357.81), and Spinal muscular atrophy – 335.1x. We were unable to assess for spinobulbar muscular atrophy/Kennedy’s disease CPT codes – 81173, 81174, 81204, as these codes were not accepted until 1/1/2019.”

2.  *Kindly re-evaluate the number of patients on "Riluzole" in your own study, and revaluate your results in comparison to other studies in the literature.*

**Response:** We re-examined our beneficiary population data at length and found no evidence of missing riluzole claims. As such, we are confident that the data in our original submission accurately reflects Medicare claims for riluzole in 2009. We reviewed the literature and we were unable to identify any other publication that provides nationwide U.S. data on use of riluzole in 2009. There are manuscripts that describe riluzole use in tertiary ALS centers, where the adherence would likely be higher due to the presence of comprehensive specialist care. We anticipate our relatively low numbers of beneficiaries for which riluzole was prescribed may be partly a reflection of an inability to assess lack of riluzole initiation due to concerns about tolerability issues or other socioeconomic factors such as cost, and a potential lack of broad dissemination of the publicly available practice parameters.

Pg #21, Line 362-367. “Furthermore, we were unable to ascertain individual provider or patient preferences for not prescribing or not receiving the practice parameters, leading to potentially perceived lower practice parameter adherence. This is a particular issue with riluzole, where medication adherence has been reported as low as 64% due to medication side effects or socioeconomic factors such as cost [49]. We also may be missing those beneficiaries prescribed riluzole who had an additional private insurance but not Medicare Part D. We anticipate this is attenuated in part, by restricting our analysis for riluzole to beneficiaries with Medicare Part D.”

3.  *Please unify the terms in the Tables within the manuscript, such as “Non-Hispanic White” and “White”, etc.*

Response: We unified the terms in the Tables within the manuscript to “Non-Hispanic White”.
