# Supplementary material for: Adherence to practice parameters in Medicare beneficiaries with amyotrophic lateral sclerosis
Source: PLoS One. 2024 Jun 3;19(6):e0304083. doi: 10.1371/journal.pone.0304083 (PMC11146737; doi:10.1371/journal.pone.0304083)
Supplement: Attachment — Submitted filename: ALS Utilization - Response to Reviewers 2.docx [file pone.0304083.s003.docx]

**PONE-D-23-13322 - Adherence to practice parameters in Medicare beneficiaries with amyotrophic lateral sclerosis.**

Dear Dr. Chidi Chima,

We appreciate the opportunity to re-revise our manuscript (PONE-D-23-13322) in response to the reviewer’s comment on our manuscript entitled “Adherence to practice parameters in Medicare beneficiaries with amyotrophic lateral sclerosis.” We have addressed the reviewer’s comment. The major change to the manuscript continues to be the inclusion of our sensitivity analysis that excludes potential amyotrophic lateral sclerosis (ALS) mimics. We excluded potential ALS mimics based on the literature, and because we chose to use the most inclusive definition of a mimic this restricted cohort was 19.7% smaller than the full cohort. This sensitivity analysis did not reveal a major difference in our findings, further strengthening our results. We have moved all methods and results from the sensitivity analysis from the Supplemental Material into the body of the manuscript, including all tables/figures to highlight these results side by side with the results from the primary analyses. We respond to the reviewer’s comment in greater detail below and mark the associated revisions in the manuscript.

In addition, we have changed the wording in the manuscripts’ main and short title from “Adherence to practice parameters in medical beneficiaries with amyotrophic lateral sclerosis” to “Adherence to practice parameters in Medicare beneficiaries with amyotrophic lateral sclerosis”. This allows us to more accurately represent our patient population and minimize any ambiguity. We have updated this in all of the documents.

**Reviewer 2**

1.  *"The author mentioned in manuscript that 19.7% of patients may have ALS-mimics. After excluding these cases, the proportion of patients using Riluzole, NIV and PEG has relatively increased. Is this increase statistically significant? If there is indeed statistical significance, would it be more reasonable to use data that excludes ALS-mimics for analysis?"*

**Response:** By definition, our more restricted ALS cohort included proportionally more patients diagnosed by a neurologist. Accordingly, as the reviewer correctly notes, this restricted cohort received more care consistent with the guidelines. However, as compared to the full cohort, the differences in the percentage who received each of the recommended interventions, which we now show in Table 2, are modest, especially when appropriately stratified by receipt of neurologist care. In addition, despite the 19.7% reduction in cohort size, there were no material changes in our findings or conclusions. Given this, and the requirement of patients to survive long enough to receive an updated diagnosis (potential for biased results), we retain analyses for both the original and restricted cohort. However, to highlight the importance of the requested restriction in the manuscript, we have moved our sensitivity analysis from the Supplemental tables 1-2 to the body of the manuscript, including in the Abstract, Methods, Results, Discussion, and all figures and tables, as detailed below. These several additions highlight the robustness of our cohort and study findings.

Abstract (Pg #2, Lines 33-35) “These associations remained marked and significant when we excluded ALS patients who subsequently received a code for other diseases that mimic ALS.”

Methods (Pg #5, Line 94-109). “As an alternative case definition for sensitivity analyses, we excluded those patients that had an ICD-9-CM code consistent with a potential ALS/MND mimic [17] after they had received a 335.2x code to minimize the possibility of inappropriately miss-accounting differences in practice parameter utilization in those patients whose diagnosis changed after the initial ALS diagnosis. These potential mimics included structural spinal pathologies (spondylosis and allied disorder – 721.0-4x, spinal stenosis in cervical region – 723.0, and spinal stenosis of other regions – 724.0x), myasthenia gravis and subtypes (358.0x, 358.1, 358.3x, 358.8, 358.9), polyneuropathies (polyneuropathy in other diseases classified elsewhere, including multifocal motor neuropathy – 357.4, diabetic polyneuropathy – 357.2, hereditary spastic neuropathy – 356.x, chronic inflammatory demyelinating polyneuropathy – 357.81), and spinal muscular atrophies (335.1x). We were unable to assess for spinobulbar muscular atrophy/Kennedy’s disease (Current Procedural Terminology [CPT] codes – 81173, 81174, 81204), as these codes were not accepted until 2019. We did not exclude patients with a code for another MND as these diseases have a common phenotype and may be difficult to fully differentiate [18]. In addition, based on prior administrative data algorithms [13], and the reported low misclassification by neurologist [17, 19, 20], we retained as true ALS cases those patients who received an ALS code from a neurologist. Utilizing this alternative case definition, we identified 6,888 (80.3%) of incident ALS cases for sensitivity analysis, representing all 3,676 ALS cases who received care from a neurologist and 3,212 of 4,899 ALS cases who did not.”

Results (Pg #7, Lines 154-156): “Our ALS cohort, with and without ALS patients who subsequently received a code for an ALS/MND mimic, had markedly shorter survival when compared to beneficiaries without an ALS code (shown in Fig 1).”

Results (Pg #12, Lines 220-223): “Finally, there was some indication that regardless of provider, riluzole use was inversely associated with distance to a multidisciplinary clinic, which strengthened in our sensitivity analyses excluding potential ALS mimics (Table 5). Otherwise, in sensitivity analyses our findings were largely unchanged when beneficiaries with potential ALS mimics were excluded from our cohort (Tables 3-5).”

Discussion (Pg #25, Lines 382-384): “Furthermore, in sensitivity analysis excluding potential ALS mimics, despite a reduction of up to 20% of our sample, our key findings were largely unchanged, highlighting the robustness of our findings and cohort.”

Tables 1 and 3-5: We have added a column of odds ratios and 95% confidence intervals from our sensitivity analysis (restricted ALS cohort), side-by-side with those from the primary analysis (full ALS cohort). There were no material changes in the findings.

Table 2 and Figure 1: We provide parallel results for the full ALS cohort and the restricted ALS cohort, and both Table 2 and Figure 1 show the similarity between the two groups. Despite a slight increment in the proportion of patients using riluzole, NIV, and PEG, there were overall no material changes in the findings, as highlighted in table 2.
